# Detection of Gelatinous Candy on Gastric Ultrasound During Preoperative Nil per Os Evaluation

**DOI:** 10.7759/cureus.101243

**Published:** 2026-01-10

**Authors:** Sathappan Karuppiah, Ratan Banik, Sudarshan Setty

**Affiliations:** 1 Anesthesiology, United Health Services, Binghamton, USA; 2 Anesthesiology, University of Minnesota, Minneapolis, USA

**Keywords:** gastric ultrasound, gummy bears, pulmonary aspiration, rapid sequence induction, survey

## Abstract

Perioperative pulmonary aspiration of gastric contents can lead to serious complications, including pneumonitis and pneumonia, which may result in significant morbidity and mortality. A 52-year-old man with a history of hypertension and diabetes presented to the hospital for emergent debridement of bilateral lower-extremity non-healing ulcers.He mentioned consuming gummy bears two hours prior to presentation, although his last substantial solid meal had occurred 14 hours earlier.Preoperatively, a gastric ultrasound revealed the presence of gummy bears within the stomach, prompting the anesthesia team to implement preventive measures to minimize aspiration risk.

We also conducted a survey to evaluate anesthesiologists' perceptions regarding the timing of patients' consumption of gummy bears before elective procedures. The survey results indicated a lack of consensus regarding the necessity of preoperative gastric ultrasound and the duration of nothing-by-mouth (NPO) status before elective procedures. Variability in skill levels and opinions among anesthesiologists regarding the optimal waiting period before surgery underscores the need for further research and standardization in these domains, highlighting the potentially critical role of gastric ultrasound in perioperative settings.

## Introduction

Pulmonary aspiration of gastric contents is a significant complication associated with general anesthesia. It is associated with substantial patient morbidity and mortality due to pneumonitis, pneumonia, sepsis, and the possible need for mechanical ventilation [1]. As part of the American Society of Anesthesiologists (ASA) "Practice Guidelines for Preoperative Fasting", patients must fast before elective surgery to ensure that their stomachs are empty prior to the induction of general anesthesia, thereby minimizing the risk of pulmonary aspiration. Depending on the type and composition of food, nothing-by-mouth (NPO) guidelines range from two to eight hours [2].

In patients with cognitive impairment and/or verbal impairment or pediatric patients, it may be challenging to obtain accurate information regarding NPO status. To address this limitation, an alternative method, ultrasound-guided assessment of gastric contents, may be considered [3]. This approach utilizes bedside ultrasound to evaluate the presence of solid or liquid contents within the stomach. Ultrasound has been shown to be reliable in detecting gastric contents and assessing aspiration risk in patients with cognitive impairment and in pediatric populations [4]. By providing objective information to healthcare providers, ultrasound-guided assessment may improve patient safety and reduce perioperative complications.

Gastric ultrasound has recently been described as a tool for estimating gastric volume and aspiration risk [5]. Its use in the preoperative setting can help guide anesthetic decision-making and may prevent unnecessary anesthesia-related risks. In this case report, we describe how preoperative gastric ultrasound influenced anesthetic management in a patient with recent gummy bear consumption.

## Case presentation

A 52-year-old man was scheduled for emergent debridement of the lower extremities due to bilateral non-healing ulcers. He presented with malodorous lower-leg ulcers accompanied by fever, chills, and signs of sepsis, including tachycardia, leukocytosis, and elevated lactate levels. His medical history was significant for hypertension and diabetes.

**Figure 1:**
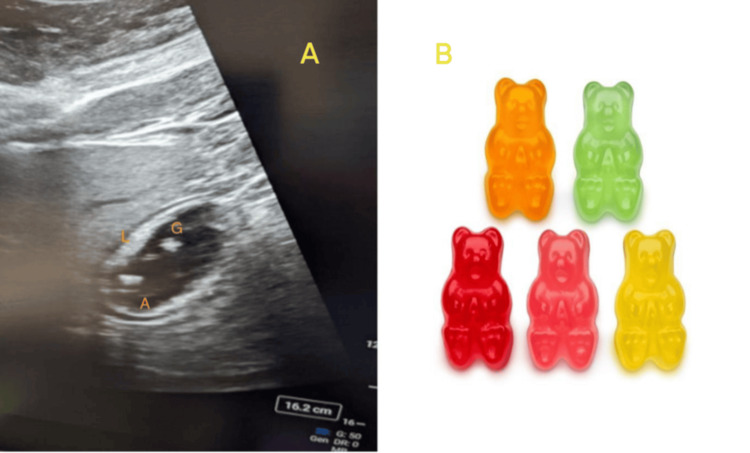
(A) Gastric ultrasound demonstrating the assessment of gastric contents. The sonographic image shows echogenic solid structures consistent with gummy bears within the stomach, along with the gastric antrum and left lobe of the liver. (B) Gummy bears L: left lobe of the liver; G: gummy bears; A: gastric antrum

During the preoperative interview, the patient reported that his last solid meal was more than 14 hours prior but that he had consumed gummy bears approximately two hours before surgery. Given his history of diabetes and recent ingestion of gummy bears, a preoperative gastric ultrasound was performed to assess aspiration risk. A curvilinear probe was placed in the epigastric region in a sagittal orientation, with the orientation marker directed cephalad. The left lobe of the liver and the gastric antrum were identified. Within the gastric antrum, two echogenic solid structures consistent with gummy bears were visualized (Figure 1).

Based on these findings, the anesthesia team elected to perform a rapid sequence induction (RSI) with cricoid pressure to minimize aspiration risk. After adequate preoxygenation, anesthesia was induced with lidocaine (1 mg/kg), propofol (1.5 mg/kg), and succinylcholine (1 mg/kg). Cricoid pressure was applied, and bag-mask ventilation was avoided. The trachea was successfully intubated using video laryngoscopy without evidence of aspiration. The intraoperative course was uneventful, and the patient was extubated at the conclusion of surgery without complications.

## Discussion

Gastric point-of-care ultrasound (POCUS) plays a critical role in assessing the volume and quality of gastric antral contents. This information is essential for determining the presence of a full stomach and estimating aspiration risk during anesthesia induction. Based on sonographic appearance, gastric POCUS allows for the qualitative assessment of gastric contents, including solid, liquid, or empty states. This information can guide anesthetic planning and determine optimal surgical timing to minimize aspiration risk.

Gummy bears are gelatin-based confectioneries composed of sugar, corn syrup, starch, flavoring, food coloring, citric acid, and, in some formulations, beeswax to prevent adhesion. They are sometimes enriched with micronutrients and used as dietary supplements, particularly in pediatric and geriatric populations [6]. The composition of gummy bears varies by brand, and differences in sugar content and additives may influence gastric emptying and aspiration risk [7]. Citric acid may increase gastric acidity, potentially exacerbating chemical pneumonitis if aspirated, while beeswax may contribute to pulmonary inflammation [7]. Therefore, consideration of gummy bear composition may be clinically relevant in perioperative risk assessment.

**Figure 2:**
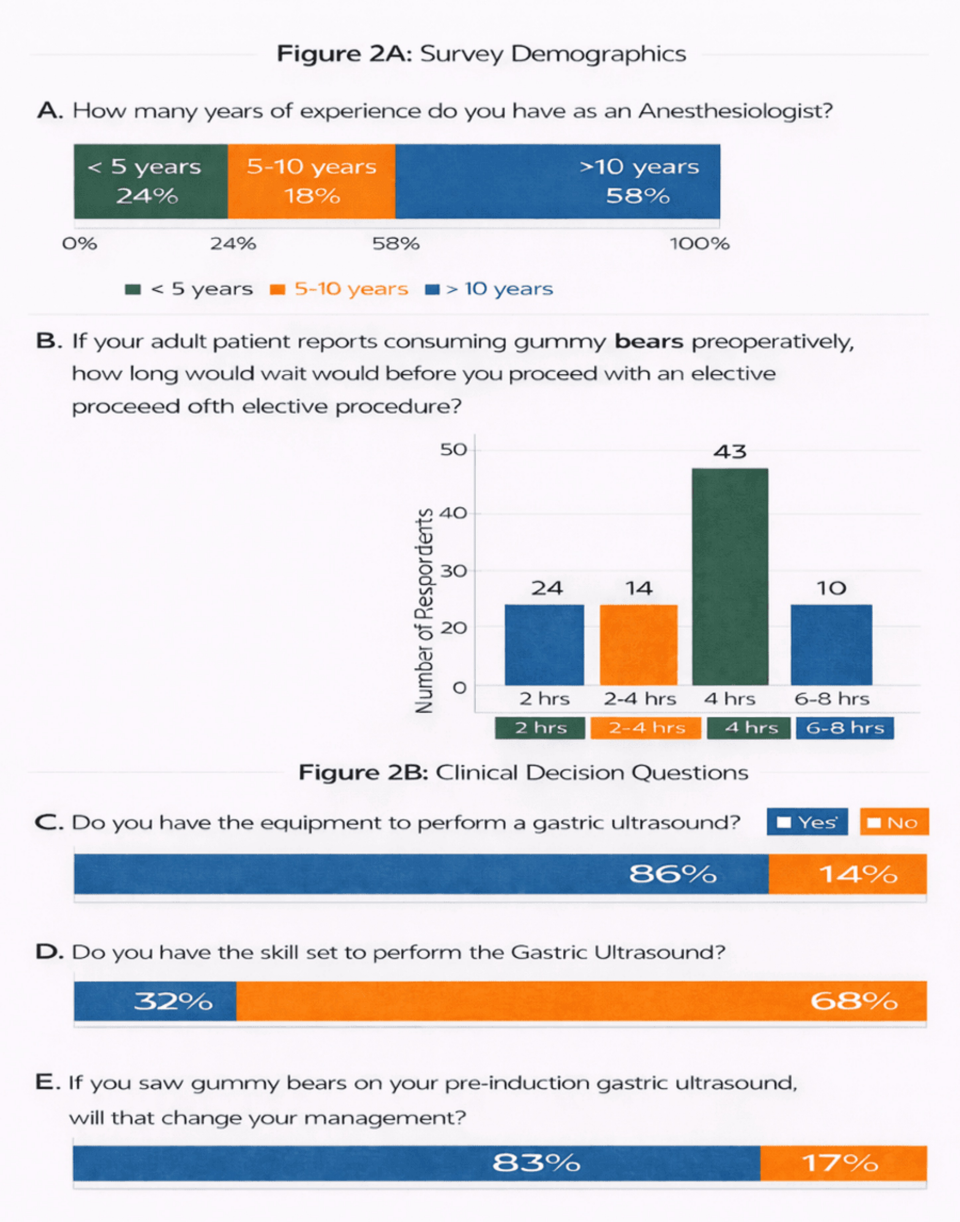
Results of the anesthesiologist survey. (A) Years of experience in anesthesiology. (B) Reported waiting period before proceeding with an elective procedure after adult patients consumed gummy bears. (C) Availability of equipment to perform preoperative gastric ultrasound. (D) Self-reported ability to perform gastric ultrasound for aspiration risk assessment. (E) Reported change in anesthetic management after the detection of gummy bears on gastric ultrasound

We conducted a survey to assess anesthesiologists' perceptions and practices regarding preoperative gummy bear consumption before elective procedures. As shown in Figure 2A-2B, the survey included 97 anesthesiologists, over 50% of whom reported more than 10 years of clinical experience. Approximately 85% reported access to equipment capable of performing gastric ultrasound; however, only 32% reported having the skills necessary to independently assess aspiration risk using this modality. This discrepancy highlights variability in training and competency related to gastric ultrasound.

Regarding anesthetic management, 83% of respondents indicated they would alter their anesthetic approach if gummy bears were identified on preoperative gastric ultrasound. With respect to surgical timing, approximately 40% (43) reported they would wait less than four hours before proceeding with anesthesia, demonstrating substantial variability in practice patterns. Overall, these findings underscore the absence of uniform consensus regarding NPO duration and the role of gastric ultrasound following gummy bear ingestion.

The ASA's updated preoperative fasting guidelines recommend not delaying surgery in healthy adults who chew gum, provided it is not swallowed [8]. However, no specific recommendations exist for gelatin-based foods such as gummy bears. While waiting 4-6 hours may be reasonable, particularly in low-risk patients, the optimal fasting duration following gummy bear consumption, especially in patients with delayed gastric emptying, remains unclear.

In this case, the presence of diabetes and recent gummy bear ingestion increased the patient's aspiration risk. Although surgery would typically be delayed in an elective setting upon the identification of solid gastric contents, the patient's evolving sepsis necessitated urgent intervention. RSI was therefore chosen to mitigate aspiration risk. While RSI can reduce aspiration risk, it is associated with potential complications, including hemodynamic instability [9,10]. Gastric ultrasound provided objective data that informed anesthetic decision-making and justified the selected approach.

Gastric ultrasound was performed to objectively assess aspiration risk. The primary purpose of gastric ultrasound is to visualize the gastric antrum, measure its cross-sectional area (CSA), and identify the presence of solid gastric contents. As described by Perlas et al. [11], a curvilinear probe is most commonly used; however, a linear probe may be appropriate in thin or pediatric patients. Gastric ultrasound is performed with the patient in both the supine and right lateral decubitus positions. The probe is placed over the epigastrium in the sagittal orientation and gently fanned from side to side to adequately visualize the antrum [12,13]. Aspiration risk is determined using a decision algorithm based on the qualitative and quantitative assessment of gastric contents. If solid (echo-dense) material is visualized or the calculated gastric volume, derived from the antral CSA, exceeds 1.5 mL/kg, the risk of aspiration is considered high [14,15].

## Conclusions

The ASA "Practice Guidelines for Preoperative Fasting" provide general recommendations for fasting durations based on food type but do not specifically address high-risk patients or gelatin-based foods such as gummy bears. Gastric ultrasound offers a personalized, objective method for assessing aspiration risk and guiding anesthetic management. Incorporating gastric ultrasound into preoperative evaluation may enhance patient safety, particularly in cases of uncertain NPO status or elevated aspiration risk.
